# Analyzing Airway Inflammation with Chemical Biology: Dissection of Acidic Mammalian Chitinase Function with a Selective Drug-like Inhibitor

**DOI:** 10.1016/j.chembiol.2011.02.017

**Published:** 2011-05-27

**Authors:** Tara E. Sutherland, Ole A. Andersen, Marie Betou, Ian M. Eggleston, Rick M. Maizels, Daan van Aalten, Judith E. Allen

**Affiliations:** 1 Centre for Immunity, Infection and Evolution, and the Institute of Immunology and Infection Research, School of Biological Sciences, University of Edinburgh, Edinburgh EH9 3JT, Scotland, UK; 2 Division of Molecular Microbiology, College of Life Sciences, University of Dundee, Dundee DD1 5EH, Scotland, UK; 3 Department of Pharmacy and Pharmacology, University of Bath, Bath BA2 7AY, England, UK

## Abstract

Acidic mammalian chitinase (AMCase) is produced in the lung during allergic inflammation and asthma, and inhibition of enzymatic activity has been considered as a therapeutic strategy. However, most chitinase inhibitors are nonselective, additionally inhibiting chitotriosidase activity. Here, we describe bisdionin F, a competitive AMCase inhibitor with 20-fold selectivity for AMCase over chitotriosidase, designed by utilizing the AMCase crystal structure and dicaffeine scaffold. In a murine model of allergic inflammation, bisdionin F-treatment attenuated chitinase activity and alleviated the primary features of allergic inflammation including eosinophilia. However, selective AMCase inhibition by bisdionin F also caused dramatic and unexpected neutrophilia in the lungs. This class of inhibitor will be a powerful tool to dissect the functions of mammalian chitinases in disease and represents a synthetically accessible scaffold to optimize inhibitory properties in terms of airway inflammation.

## Graphical abstract

**Graphical Abstract:**

**Figure:**
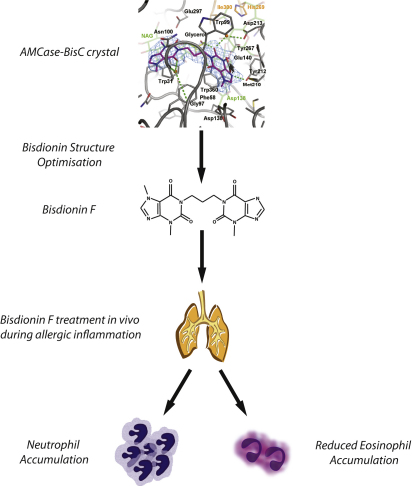

**Highlights:**

► A novel chitinase inhibitor was designed guided by the AMCase crystal structure ► BisF inhibits AMCase activity with 20-fold selectivity over chitotriosidase ► BisF shows efficacy in vivo in a murine model of airway inflammation ► BisF treatment revealed new functions for AMCase during allergic lung inflammation

## Introduction

Chitin, the second most abundant polysaccharide in nature, is a principal component of the arthropod exoskeleton, nematode eggshell, and fungal cell wall. Although mammals themselves do not synthesize chitin, they are continually exposed to this polymer through inhalation and exposure to chitin-containing pathogens. Chitin accumulation is limited through hydrolysis of β(1→4) glycosidic bonds by chitinases, members of the evolutionary conserved glycoside hydrolase family 18 (GH18). Mammals have two genes encoding active chitinases, chitotriosidase (CHIT1) and acidic mammalian chitinase (AMCase), that represent an ancient gene duplication event and show sequence homology to bacterial chitinases (Bussink et al., 2007). More recent gene duplications have yielded the homologous chitinase-like proteins (CLPs) with mutations within the enzymatic machinery rendering the catalytic site inactive (Zaheer-ul-Haq et al., 2007). Although the functions of both chitinases and CLPs in mammals are still poorly understood, it is becoming clear that their expression is regulated in both innate and adaptive immune responses. CHIT1, which is expressed exclusively in phagocytes (Boot et al., 2005), is thought to play an important role in the mammalian innate immune response against fungi, bacteria, and other pathogens (Barone et al., 2003; Labadaridis et al., 2005). Conversely, increased production of AMCase and CLPs Ym1, Ym2, and BRP-39 in rodents and YKL-39 and YKL-40 in humans is a prominent feature of Th2-driven pathologies, including infection, allergic inflammation, and asthma (reviewed in Sutherland et al., 2009).

AMCase was first described to be expressed in the gastrointestinal tract and lungs of rodents and humans (Boot et al., 2001). AMCase is expressed in tissue macrophages and epithelial cells, with its production driven by Th2-cytokines IL-4 and IL-13 (Zhu et al., 2004). Early exploration of mammalian chitinase function implicated AMCase as a mediator of Th2-driven allergic airway diseases following the use of the chitinase inhibitor allosamidin, a pseudotrisaccharide natural product derived from *Streptomyces* species (Sakuda et al., 1986), in murine models (Zhu et al., 2004). Treatment of allergen-challenged mice with allosamidin or demethylallosamidin significantly reduced eosinophilia, a hallmark of allergic inflammation (Matsumoto et al., 2009; Zhu et al., 2004). Although both compounds inhibit chitinase activity in vivo*,* only demethylallosamidin treatment reduces allergen or IL-13-induced airway hyperresponsiveness. Despite beneficial actions in models of Th2-driven allergic inflammation, the therapeutic potential of these compounds is limited due to their expensive and complex synthesis and commercial unavailability. In addition, allosamidin has a broad range of activity against all family 18 chitinases (Berecibar et al., 1999) and possesses physicochemical properties that are not compatible with a drug-like compound, such as high molecular weight (604.7 Da), an undesirably low clogP (−4.7), and poor ligand efficiency (−0.25 kcal·mol^−1^·atom^−1^ for fungal chitinase) (Vaaje-Kolstad et al., 2004). Allosamidin is a more effective inhibitor of CHIT1 than AMCase (IC_50_ murine CHIT1 [mCHIT1] ∼50 nM and murine AMCase [mAMCase] ∼400 nM) (Zheng et al., 2005; Boot et al., 2001). This is of particular concern as CHIT1 is not an effector molecule in allergic inflammation and is rather regarded as a host-defense mechanism against chitin-containing pathogens (reviewed in Sutherland et al., 2009). Thus, there is a need to identify compounds that are drug-like selective inhibitors of AMCase that can be used in animal models to dissect the roles of the chitinases in allergic airway inflammation and potentially further develop as anti-asthma therapies.

We recently identified xanthine derivatives as promising leads for GH18 inhibitors (Rao et al., 2005) and subsequently developed a low micromolar chitinase inhibitor composed of two linked caffeine molecules (bisdionin) with desirable drug-like properties, a crystallographically defined binding mode, and excellent synthetic accessibility (Schuttelkopf et al., 2006). Here, we describe the rational design of a novel AMCase inhibitor, bisdionin F, with 20-fold selectivity for AMCase over CHIT1 and demonstrate in vivo activity in a mouse model of acute allergic inflammation. Bisdionin F treatment in allergen-challenged mice reduced eosinophil recruitment and measurements of ventilatory function. Unexpectedly however, treatment with bisdionin F also resulted in neutrophilia and changes to expression of genes associated with remodeling. These studies highlight the complex mechanistic pathways surrounding the therapeutic inhibition of AMCase activity. Nonetheless, the potent selective activity of bisdionin F in vitro and in vivo and its relatively easy synthesis makes this inhibitor an invaluable tool for the chemical biological dissection of the roles of the different mammalian chitinases.

## Results

### Rational Design of Bisdionin F, a hAMCase Selective Inhibitor

**Figure 1:**
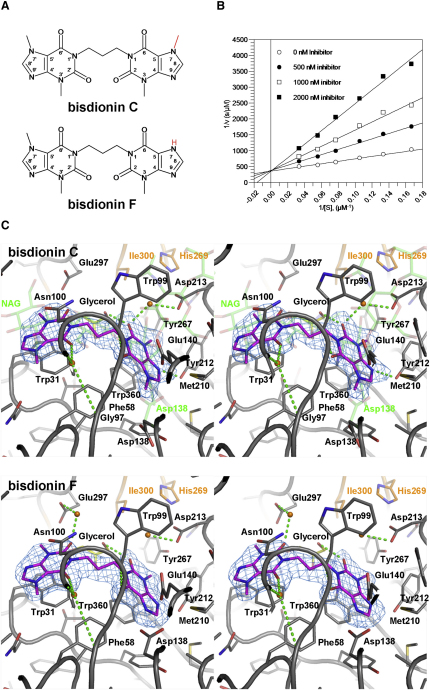
Activity of Bisdionin F and a Structural Comparison of Bisdionin-hAMCase Complexes (A) Chemical structures and atom numbering of bisdionin C and bisdionin F are shown with the differing methyl/hydrogen moieties highlighted in red. (B) Lineweaver-Burk plot showing bisdionin F inhibition of hAMCase at different concentrations. The data are compatible with a competitive inhibition model, giving a K_i_ of 420 ± 10 nM. (C) Stereo figures of the active sites (monomer A) of the hAMCase-bisdionin C (top) and hAMCase-bisdionin F (bottom) complexes. Unbiased Fo-Fc φ_c_ electron density maps are contoured at 2.5σ. Protein side chains, glycerol, and ligand molecules are shown as a stick models with gray, yellow, and magenta C atoms, respectively. hAMCase residues not conserved compared to CHIT1 are shown with orange C atoms. Water molecules interacting with the ligand are shown as orange spheres and hydrogen-bonding interactions are shown as dotted green lines. The second, less-defined, ligand molecules stacking against Trp99 and Trp218 are omitted for clarity. N-acetyl glucosamine residues taken from the HCGP-39 in complex with chitin (PDB ID 1HJW) and Asp138 in the “up-conformation” are shown as stick models with transparent green C atoms.

**Figure 2:**
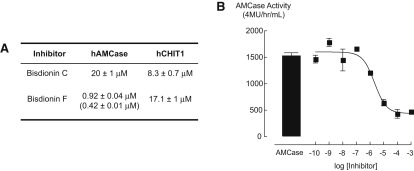
Chitinase Inhibition by Bisdionin Compounds (A) IC_50_ values (K_i_ values are shown in parenthesis) of bisdionin C and F compounds against hAMCase and hCHIT1. All values are given in micromolar. (B) Bisdionin F inhibits recombinant mAMCase enzymatic activity in vitro. Chitinase activity was determined using a fluorescent 4-methylumbelliferyl (4-MU) substrate. An AMCase expressing COS-7 cell-free supernatant was used as a source of enzymatically active recombinant mAMCase. rAMCase, in the presence of 4-MU substrate, was incubated with DMSO vehicle or increasing concentrations of bisdionin F for 10 min at 37°C. Chitinase activity is measured relative to the amount of substrate hydrolysed/hr/mL sample. IC_50_ = 2.21 ± 0.18 μM.

A recent report described the reduction of airway eosinophilia upon inhibition of total bronchoalveolar chitinase activity with the natural product chitinase inhibitor allosamidin (Zhu et al., 2004). We recently described the bisdionins, dixanthine derivatives that are micromolar inhibitors of family 18 chitinases (Schuttelkopf et al., 2006). A high-resolution crystal structure of bisdionin B (C2-dicaffeine) complexed with *Aspergillus fumigatus* chitinase B1 (*Af*ChiB1) was solved and revealed the binding mode of bisdionin B (Schuttelkopf et al., 2006). While being less energetically favorable, the caffeine linker length of these molecules could be modified to alleviate strain and result in a more potent inhibitor (Schuttelkopf et al., 2011). The most potent of these, bisdionin C (Figure 1A), is a drug-like molecule as assessed by Lipinski's rule of five: it has six hydrogen bond acceptors and no hydrogen bond donors, a molecular weight of 400.4 Da, a clogP of approximately 0, and a ligand efficiency of −0.41 kcal·mol^−1^·atom^−1^ against *Af*ChiB1 (Schuttelkopf et al., 2011). We investigated whether bisdionin C would inhibit human AMCase (hAMCase) and/or human chitotriosidase (hCHIT1). Assessment of chitinase activity using a fluorescent substrate revealed that while bisdionin C inhibits hAMCase and hCHIT1 in the micromolar range, it does so with no apparent selectivity (Figure 2A).

To facilitate structure-guided optimization of the bisdionin scaffold into a potent, selective hAMCase inhibitor, the crystal structure of the hAMCase-bisdionin C complex was determined to 2.2 Å resolution (Table 1 and Figure 1C). The native structure of hAMCase has recently been reported (Olland et al., 2009), giving an rmsd of 0.80 Å with the structure reported here. The loops on the AMCase TIM barrel [(βα)_8_ fold] produce a deep active site cleft similar to other “bacterial-type” family 18 chitinases. Bisdionin C spans the −1, −2, and −3 GlcNAc binding subsites of the AMCase chitooligosaccharide substrate (Figure 1C). The methyl xanthine units bind at the bottom of the active site, stacking on the indole groups of Trp31 and Trp360 (Figure 1C). The hydroxyl group of Tyr212 forms a hydrogen bond with N9, whereas the backbone N of Trp99 forms a hydrogen bond with O6. Water-mediated hydrogen bonds are formed between the carboxyl group of Asp213 and O2 and between the backbone oxygen and nitrogen atoms of Gly97 and Phe101, respectively, and bisdionin C O6′.

Although hCHIT1 and hAMCase catalytic domains share 57% sequence identity, there are two amino acids near the catalytic machinery that are different in hAMCase, His269 (Arg269 in hCHIT1) and Ile300 (Met300 in hCHIT1) (Figure 1C). Interestingly, the N7 methyl group of bisdionin C appears to impose an unfavorable conformation of Asp138, a key catalytic residue that hydrogen bonds the catalytic acid (Glu140)/substrate *N*-acetyl group and stabilizes the oxazolinium ion reaction intermediate during catalysis (Brameld et al., 1998; Terwisscha van Scheltinga et al., 1995; van Aalten et al., 2001) (Figure 1). Given the unfavorable interactions of the N7 methyl group and the nonconserved amino acid substitutions on the opposite side of the xanthine moiety, we explored the effects of the N7 methyl group on potency and selectivity. We synthesized bisdionin F, the N7-demethylated derivative of bisdionin C (Figure 1A). A 2.25 Å crystal structure of the hAMCase-bisdionin F complex reveal that Asp138 now adopts the “up” conformation, generating an additional hydrogen bond with the N7 of the xanthine in the −1 subsite, and also interacting with the catalytic acid (Glu140). The inhibitor bisdionin F was shown to further increase hAMCase inhibition by over one order of magnitude compared to bisdionin C, competitively inhibiting the enzyme with a *K*_i_ = 420 ± 10 nM (Figure 1B). The inhibitor shows this improved inhibition only toward hAMCase, not hCHIT1 (IC_50_ = 17 μM), thus introducing 20-fold selectivity (Figure 2A). It should be noted that hAMCase possesses a more negatively charged active site, generated by the Arg269 (hCHIT1) to His269 (hAMCase) substitution, also lowering the pH optimum of the enzyme. Thus, electrostatic effects may explain why the imidazole moiety, generated by removing the methyl group, is better accommodated by the hAMCase enzyme.

### Bisdionin F Reduces Chitinase Activity in a Murine Model of Allergic Inflammation

**Figure 3:**
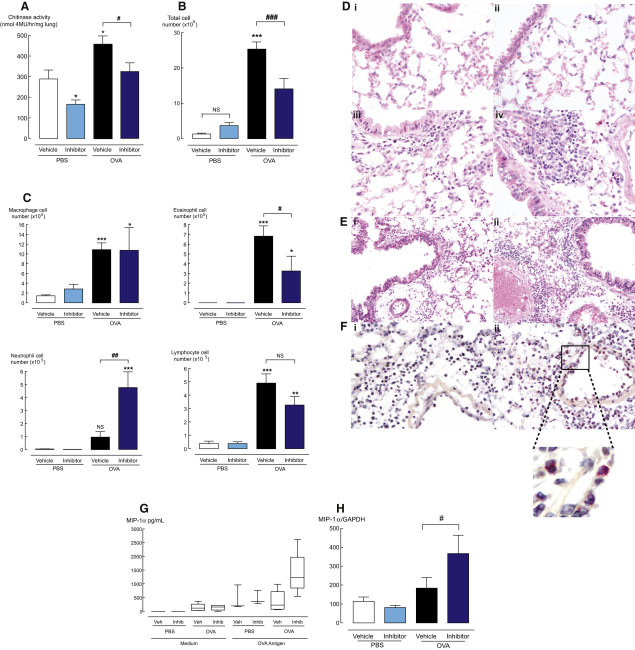
Inhibition of Allergen-Induced Lung Chitinase Activity and Changes to Cell Recruitment in Lavage and Lung Tissue (A) Chitinase activity measured in lung homogenates from PBS/OVA-challenged mice treated with vehicle or bisdionin F. Bisdionin F reduced chitinase activity (expressed as 4MU substrate hydrolysed/hr/mg of lung tissue) in both PBS-challenged and OVA-challenged mice. (B and C) Total cell number (B) and differential cell counts (C) of macrophages, lymphocytes, neutrophils, and eosinophils in bronchoalveolar lavage from bisdionin F and vehicle treated PBS and OVA-challenged mice. (D) Representative H&E stained lung sections (i) Vehicle PBS-challenged mice, (ii) bisdionin F PBS-challenged mice, (iii) vehicle OVA-challenged mice, and (iv) bisdionin F OVA-challenged mice. Magnification, ×400. (E) Representative H&E stained lung sections from a second independent experiment; (i) vehicle OVA-challenged mice and (ii) bisdionin F OVA-challenged mice. Magnification, ×200. (F) Representative lung sections stained with naphthol AS-D choloracetate esterase showing (i) vehicle OVA-challenged mice or (ii) bisdionin F OVA-challenged mice. Neutrophils exhibit red staining; cell nuclei stained with hematoxylin. Magnification, ×400. (G) Protein levels of MIP-1α in supernatant of tLN cells from PBS or OVA-challenged mice, cultured in RPMI or OVA antigen (0.5 mg/mL) for 72 hr. (H) Expression of MIP-1α mRNA from lung of PBS and OVA-challenged mice, normalized to the level of housekeeping gene, GAPH, in individual lung samples. Chitinase inhibitor, bisdionin F, 5 mg/kg i.p., n = 5–7 per group. NS, not significant. ^∗^p < 0.05, ^∗∗^p < 0.01, and ^∗∗∗^p < 0.001 compared to vehicle PBS; #p < 0.01, ##p < 0.01, ###p < 0.001. Data is representative of three individual experiments.

To verify that, as expected, bisdionin F would have similar activity against the mouse enzyme, recombinant mAMCase was stably expressed in COS-7 cells. After 10 min incubation, bisdionin F treatment resulted in a concentration-dependent inhibition of mAMCase activity with an IC_50_ of 2.2 ± 0.2 μM (Figure 2B). To test the in vivo efficacy of bisdionin F, a well-established model of airway lung inflammation was used, in which mice are first sensitized with ovalbumin (OVA) i.p. and then challenged in the airways, leading to increased chitinase activity in the lung tissue (Zhu et al., 2004). Enzymatic activity in lung homogenates of mice treated with 5 mg/kg bisdionin F (Figure 3A) was assessed in this model. As previously reported, chitinase activity significantly increases upon allergic challenge, as assayed approximately 24 hr after the last challenge, while treatment with bisdionin F significantly reduced chitinase activity in the lungs of both control PBS and OVA-challenged mice.

### Bisdionin F Modulates Allergen-Induced Inflammation

To assess the impact of AMCase inhibition on allergen-induced inflammation, cellular infiltrate into the bronchoalveolar lavage fluid (BALF) was examined on cytospins from vehicle and bisdionin F-treated animals (Figures 3B and 3C). As expected, acute OVA challenge induced a significant increase in eosinophils, lymphocytes, and macrophages in the lavage fluid compared to PBS-challenged mice. Strikingly, bisdionin F-treated allergic mice were found to have significantly reduced total cell airway infiltrates (Figure 3B, p < 0.01 compared to vehicle treatment), whereas cell numbers in PBS-challenged animals were not altered with chitinase inhibition. Differential counts of cells recovered from the BALF revealed a reduction in the number of lymphocytes and eosinophils following chitinase inhibition (Figure 3C). However, the most unanticipated result of bisdionin F treatment was a 4-fold increase in neutrophil cell number compared to vehicle-treated OVA-challenged mice (Figure 3C).

Changes to inflammatory infiltrates were examined in hematoxylin- and eosin-stained lung sections (Figures 3D and 3E). PBS-challenged mice had similar lung structure and cellular composition, whether treated with bisdionin F or vehicle (Figures 3Di and 3Dii). Allergen challenge resulted in inflammatory cell influx into the lamina propria, perivascular, and peribronchiolar regions of the lung. Following treatment with bisdionin F in allergic animals, inflammatory influx into the lung tissue was more striking (Figures 3D and 3E). Staining with naphthol AS-D choloracetate esterase, a stain specific for neutrophil granulocytes, revealed predominant neutrophil influx in bisdionin F OVA-challenged mice (Figure 3F), consistent with the analysis of the BALF (Figure 3C).

To investigate the cause of the bisdionin F-induced neutrophilic response, cytokine and chemokine secretion from OVA-specific tLN cell cultures were examined with Luminex multiplex bead array. Potent neutrophil chemotactic factors KC (murine IL-8 equivalent) and IL-17 were not significantly altered in tLN cultures from chitinase inhibitor treated allergic mice (data not shown). However, both the secretion and expression of chemokine macrophage inhibitory protein-1 alpha (MIP-1α), also a neutrophil chemoattractant, were enhanced by bisdionin F treatment in OVA-challenged animals (Figures 3G and 3H). MIP-1α levels were not altered by OVA-challenge alone, correlating with a lack of significant neutrophil recruitment in these mice (Figure 3C).

### Altered Eosinophil Recruitment Following Bisdionin F Treatment Is Dose Dependent

**Figure 4:**
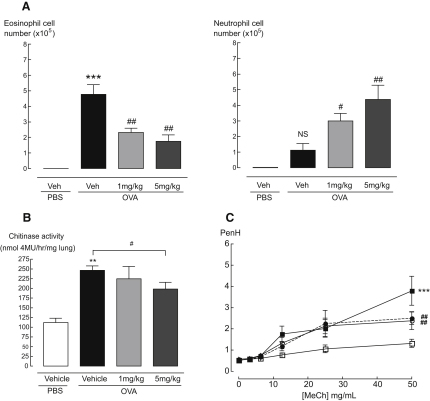
Treatment with Two Different Doses of Bisdionin F during Allergic Inflammation (A) Eosinophil and neutrophil cell numbers in the bronchoalveolar lavage from chitinase inhibitor treated allergic mice compared to vehicle treatment. (B) Chitinase activity measured in lung homogenates from OVA-challenged mice treated. (C) Relationship of chitinase inhibition on ventilatory function in allergic animals. PenH values were measured in conscious, unrestrained mice administered with increasing doses of an aerosolized bronchoconstrictor, methacholine. Vehicle-treated PBS-challenged mice, open squares; Vehicle-treated OVA-challenged mice, closed squares; 1 mg/kg bisdionin F OVA-challenged, open circles; 5 mg/kg bisdionin F, closed circles, dashed line. Chitinase inhibitor, bisdionin F 1 and 5 mg/kg, i.p.; n = 5–6 mice per group. NS, not significant. ^∗∗^p < 0.01 and ^∗∗∗^p < 0.001 compared to vehicle PBS and #p < 0.05 and ##p < 0.01 compared to vehicle OVA-challenged mice.

At a dose of 5 mg/kg, bisdionin F decreased eosinophil cell number and increased neutrophil cell number, resulting in an unfavorable cell recruitment profile for the treatment of allergy. Thus, we investigated whether a lower dose of bisdionin F would allow effects on neutrophil and eosinophil cell numbers to be segregated. The lowest dose at which we could observe any chitinase inhibition was 1 mg/kg, and thus allergic animals were treated with 1 and 5 mg/kg of bisdionin F and eosinophil and neutrophil recruitment was assessed (Figure 4A). Increases in eosinophilia of OVA-challenged mice were reduced by treatment with both 1 and 5 mg/kg dose. However, at both doses, bisdionin F treatment also resulted in a significant 2- to 4-fold increase in neutrophil cell number. A bronchoconstrictor, methacholine, was administered following challenge with OVA or PBS to measure penH (enhanced pause), a measurement that reflects changes to ventillatory function in spontaneously breathing mice, as described in detail in the Experimental Procedures. As expected, penH was significantly increased in vehicle-treated allergic animals compared to naive animals (p < 0.001, Figure 4C). A dose of 5 mg/kg bisdionin F had no effect on penH measurements in PBS-challenged animals (data not shown). However, bisdionin F treatment significantly reduced penH in allergic mice at both 1 and 5 mg/kg at the highest concentration of methacholine used.

### Expression of Genes Associated with Tissue Remodeling Are Altered by Chitinase Inhibition

**Figure 5:**
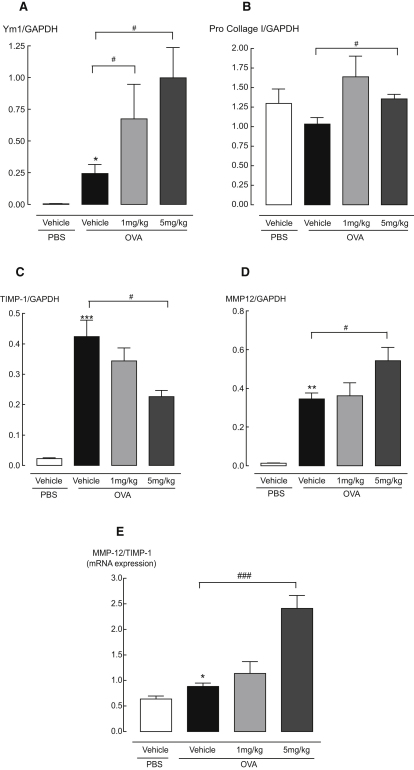
Chitinase Inhibition Alters the Expression of Tissue Remodeling Genes (A–D) Chitinase inhibition alters the expression of genes implicated in lung tissue remodeling. mRNA expression of (A) Ym1, (B) pro-collagen I, (C) TIMP-1, and (D) MMP-12 were measured in RNA extracted from lung tissue of PBS-challenged animals or vehicle and bisdionin F-treated allergic animals. (E) The ratio of MMP-12:TIMP-1 mRNA expression. Chitinase inhibitor, bisdionin F, 1 and 5 mg/kg i.p.; n = 5–6 mice per group. ^∗^p < 0.05, ^∗∗^p < 0.01, and ^∗∗∗^p < 0.001 compared to vehicle PBS-challenged mice; #p < 0.05 and ###p < 0.001 compared to vehicle OVA-challenged mice.

It has been suggested that chitinases play a role in tissue remodeling responses in models of infection and Th2-driven inflammation (reviewed in Lee 2009) with eosinophils also implicated in remodeling. We thus predicted that chitinase inhibition, leading to reduced eosinophilia, might have beneficial effects on the expression of genes associated with lung remodeling. Contrary to our expectation, the expression of genes associated with remodeling including procollagen I, matrix-metalloprotease-12 (MMP-12), and Ym1 (chitinase-like protein) were significantly increased in bisdionin F-treated animals, while the tissue inhibitor of metalloproteinases 1 (TIMP-1) was downregulated (Figures 5A-D). Furthermore, the ratio of MMP:TIMP expression was enhanced 2.5 fold following bisdionin F treatment, suggesting enhanced MMP activity is likely (Figure 5E).

## Discussion

The therapeutic targeting of chitinase enzymatic activity was proposed when it was discovered that AMCase is highly expressed in both animal models of allergic inflammation and in human asthmatics (Bierbaum et al., 2005; Zhu et al., 2004) and that nonspecific inhibition of chitinases had anti-inflammatory effects (Matsumoto et al., 2009; Zhu et al., 2004). However, the inhibitors used in these studies are not specific for AMCase and do not provide tangible starting points for the development of such compounds. With the aid of hAMCase structural data, we undertook the design of a selective hAMCase inhibitor that would allow us to more precisely dissect the role of AMCase in allergic inflammation. The design strategy for the novel chitinase inhibitor bisdionin F demonstrates that selective inhibitors of AMCase activity can be synthesized and as shown here, used in vivo to examine the function of AMCase during Th2-driven allergic inflammation. Importantly, our findings suggest that key properties of AMCase may have been overlooked using broad chitinase inhibitors.

Bisdionin F showed micromolar affinity against recombinant mAMCase in vitro and reduced both the increased lung chitinase enzymatic activity induced by allergic OVA challenge and the basal level of chitinase activity in naive mice. Treatment with bisdionin F significantly reduced eosinophil cell numbers in the lavage of allergic mice, an effect that has been previously described for other chitinase inhibitors (Matsumoto et al., 2009; Zhu et al., 2004). Although the central role of eosinophils in the allergic reaction is sometimes debated, reduced eosinophil numbers are associated with improvements in ventilatory function and tissue remodeling (Flood-Page et al., 2003; Humbles et al., 2004). In this current study, chitinase inhibition decreased penH in allergic animals at doses in which eosinophil recruitment was reduced by approximately 50%, supporting the notion that eosinophils regulate ventilatory function.

The most striking and consistent feature of bisdionin F treatment was the neutrophil recruitment observed in OVA-challenged mice but not control PBS-challenged mice. While not considered a classical inflammatory mediator in Th2-driven allergy, neutrophils have increasingly and controversially been placed in the spotlight as important mediators of persistent and corticosteroid-resistant asthma (Green et al., 2002; Jatakanon et al., 1999). Recent studies have correlated chronic asthma severity with the numbers of neutrophils in the sputum and bronchial biopsies (Louis et al., 2000; Woodruff et al., 2001) with neutrophil recruitment and activation mediated largely by IL-8 (Monteseirin 2009). Bisdionin F-induced neutrophilia was accompanied by an increase in MIP-1α secretion and expression, both at the site of inflammation and the draining lymph nodes. Although the role of MIP-1α during allergic asthma has been described to a lesser extent than IL-8, the levels of MIP-1α are increased in lavage fluid from allergic asthmatics (Alam et al., 1996) and hence may be an important component for induction of neutrophil chemotaxis. In addition to increased neutrophil numbers following bisdionin F treatment, we observed alterations in airway remodeling genes that would be predicted to have negative consequences for lung function. Whether these changes were the result of the altered eosinophil/neutrophil balance or a more direct effect of the inhibitor remains to be determined.

Treatment with demethylallosamidin did not result in neutrophil recruitment in allergic mice (Matsumoto et al., 2009), while the effects of allosamidin and anti-AMCase sera on neutrophil cell number were not reported (Zhu et al., 2004). Bisdionin F-induced neutrophilia does correlate well with the inhibition of chitinase activity. Furthermore, in previous work, the potential side effects of xanthine-based (bisdionin) chitinase inhibitors were explored by monitoring phosphodiesterase inhibition, a known target of xanthine derivatives (Rao et al., 2005). Results showed that as larger substituents were added to the N1 position of the xanthine structure, selectivity for the chitinases increased. Bisdionin F is further extended at this position, reducing the likelihood of off-target effects, although these cannot be fully excluded. Bisdionin F-induced neutrophilia could be mediated, at least in part, through chitin accumulation in the lungs. Chitin has been shown to induce inflammatory cell recruitment (Reese et al., 2007), including neutrophils (Da Silva et al., 2008). While these immunological actions of chitin would normally be limited in mammals by chitinase-mediated chitin degradation, interference with chitinase enzymatic activity would likely result in chitin accumulation. Because bisdionin F exhibits selectivity for AMCase, unlike allosamidin, which is more effective at inhibiting CHIT1 (Boot et al., 2001; Zheng et al., 2005), the activity of CHIT1 in the lung should remain largely unaffected. Both CHIT1 and AMCase may be required to ensure full degradation and clearance of chitin. The level of chitinase activity in the lung and the predominance of one enzyme over the other may influence the size and quantity of chitin degradation products, which has been shown to determine the inflammatory outcome (Da Silva et al., 2008). Importantly, if the ability of AMCase to break down chitin is important, the absence of neutrophils in PBS-challenged mice treated with bisdionin F suggests that other factors are at play and that an actively primed immune environment is required for chitin to induce neutrophilia.

In addition to inhibiting chitinase activity (Matsumoto et al., 2009; Zhu et al., 2004), AMCase has been targeted by RNA interference (Yang et al., 2009) and anti-AMCase-sera (Zhu et al., 2004). The overlap of all three treatments appears to be a reduction in eosinophilia, also observed with bisdionin F. Approaches that more specifically targeted AMCase yielded additional effects not seen with broad chitinase inhibitors, including reduction of IL-13-induced chemotactic factors, antigen-specific IgE responses, and airway hyperresponsiveness. Also consistent with our study, the RNA interference led to a small increase in neutrophils in animals infected with an adenoviral expressing short hairpin RNA (shRNA) against AMCase relative to mice that received a shRNA control (Yang et al., 2009). Both anti-AMCase and shRNA treatment are likely to have influenced protein levels and thus will not solely have addressed the role of AMCase enzymatic activity.

These studies, along with the findings presented here, emphasize the importance of generating specific tools for dissecting the role of chitinases during Th2-driven allergic inflammation. A recent study has developed high-throughput-, fragment-, and virtual-based screening methods to identify a selective inhibitor of AMCase activity (Cole et al., 2010). The study demonstrated inhibition of chitinase activity in vivo, albeit, at a much greater dosing regime (50 mg/kg twice daily) compared to bisdionin F, but did not investigate the immunological or physiological consequences. We have used both structural and enzyme inhibition data to successfully design bisdionin F and utilized this compound in vivo to selectively inhibit AMCase chitinolytic activity during allergic airway inflammation. While our study has raised important questions regarding the therapeutic benefit of chitinase inhibition for the treatment of Th2-driven inflammatory conditions, bisdionin F is a valuable tool for understanding the yet unknown functions of AMCase. Further, studies in which the active site Asp138 has been mutated to Ala have demonstrated distinct enzyme-dependent and -independent properties for AMCase that can both be blocked by allosamidin (Hartl et al., 2009). Thus, development of therapeutically useful inhibitors may still be possible, based on further refinement of the bisdionins in conjunction with a better understanding of both the chitinases and CLPs, some of which, like mutant AMCase, can still bind chitin (and thus presumably chitinase inhibitors), but cannot cleave it (Hartl et al., 2009; Mohanty et al., 2009).

The active chitinases are highly conserved across mammals, while the CLPs represent more recent gene duplication events with subsequent loss-of-function mutations (Bussink et al., 2007). This has resulted in an intriguing situation in which all mammals express the highly conserved active enzymes chitotriosidase and AMCase but additionally express a broad range of diverse CLPs without known function. The data presented herein have already demonstrated novel inhibitory effects of AMCase on neutrophil recruitment potentially through MIP-1α signaling. Intriguingly, following the direct transfection of Ym1 (a murine CLP) into the lungs of naive mice, we have observed neutrophil recruitment and enhanced MIP-1α secretion (data not shown). Thus, enhanced Ym1 expression following bisdionin F treatment (Figure 5A) may explain the increases in MIP-1α and neutrophilia. This raises the exciting possibility that chitinases and CLPs have cross-regulatory properties. Further, the dissection of the differential roles of this expanded gene family may lead to future combination therapies in which both eosinophilia and neutrophilia can be repressed for the successful treatment of allergies.

Although mouse CLPs cannot fully represent the human proteins, the evolutionary principles driving the remarkably rapid divergence of CLPs are likely to be shared across species. Thus studies in mice should allow us to address fundamental functional differences between chitinases and CLPs. Indeed, the potential capacity of broad chitinase inhibitors such as allosamidin to bind a range of CLPs may have previously obscured AMCase-specific activities. It is only through the use of selective chemical tools like the bisdionins that we can begin to unravel the complex mechanistic and regulatory pathways of chitinase and CLP functions.

## Significance

**Chitotriosidase (CHIT1) and acidic mammalian chitinase (AMCase) are mammalian chitinases found in the lung and are upregulated during innate and adaptive immune responses, respectively. AMCase has previously been identified as a mediator in allergic inflammation and asthma, although most information regarding AMCase function has been provided through studies using allosamidin, a nonspecific inhibitor of family 18 chitinases. To address the role of AMCase during Th2-driven inflammation, we used a rational approach to design a selective inhibitor of AMCase chitinase activity, bisdionin F. Bisdionin F showed in vivo efficacy in a murine model of allergic inflammation and, similar to allosamidin, attenuated lung chitinase activity, reduced eosinophil influx, and improved ventilatory function. However, our studies with bisdionin F reveal functions of AMCase that have previously gone unreported, likely due to the unspecific nature of other chitinase inhibitors. Neutrophils, while not typically associated with a Th2-allergic response, were strikingly enhanced with AMCase inhibition. While such results question the therapeutic potential of bisdionin F monotherapy and, indeed, other chitinase inhibitors for Th2-inflammatory conditions, it does not preclude the possibility to design AMCase inhibitors with appropriate actions. For example, beneficial effects of allosamidin and bisdionin F may be due to actions that are independent of direct chitinase activity. This same class of inhibitor could potentially be developed with activity against chitinase-like proteins (CLPs) without affecting chitinase activity. Thus, an understanding of the actions of the highly diverse CLP family, which are also upregulated during Th2-driven conditions, as well as enzyme-independent actions of AMCase, warrants urgent attention. Overall, the approach of designing a specific class of inhibitor that shows selectivity for AMCase has provided an invaluable tool to begin dissecting the function of AMCase during pathology and has already alluded to the potential of cross-regulatory actions of the chitinase and CLP family members.**

## Experimental Procedures

### Bisdionin Synthesis

Bisdionin C (Itahara and Imamura 1994), with an alkyl linker of three methylene units, was synthesized as previously described (Schuttelkopf et al., 2011). Bisdionin F was prepared according to the method of Allwood et al. (Allwood et al., 2007) by the alkylation of 1-(3-bromopropyl)-3, 7-dimethyl-1*H*-purine-2,6(*3H*, 7*H*)-dione (Fischer et al., 1999) with 7-(4-methoxybenzyl)-3-methyl-1*H*-purine-2,6(3*H*, 7*H*)-dione (Sakai et al., 1992), followed by removal of the 4-methoxybenzyl group under acidic conditions (Sakai et al., 1992). Compounds were characterized by ^1^H and ^13^C NMR and HRMS and revealed no trace contamination by high molecular weight species such as LPS. Purity was > 95% as judged by analytical HPLC.

### Protein Expression, Crystallization, and Structure Determination of hAMCase Complexes

A fragment corresponding to hAMCase 22-398 (bp 64–1194) was ligated into the pPIC9 vector (Invitrogen) using the Xho I and Not I restriction sites. The enzyme was subsequently overexpressed as a secreted protein from the *Pichia pastoris* GS115 strain and purified using a combination of affinity chromatography and size-exclusion chromatography. Pure hAMCase was spin concentrated to 37 mg/ml in 25 mM HEPES (pH 6.8), 250 mM NaCl. The protein was crystallized at 30°C from 75% saturated NaCl, 0.1 M HEPES (pH 7.4) using the hanging drop method. Crystals grew to an approximate size of 200 × 100 × 50 μm. Crystals were cryoprotected in 50% saturated NaCl, 20% glycerol in 0.1 M HEPES (pH 7.4) and subsequently flash frozen in liquid nitrogen. The binary complexes were formed by soaking crystals in reservoir solution containing saturated concentrations of bisdionin C (4 hr) and bisdionin F (2 hr) prior to cryoprotection. Data for hAMCase were collected at ID14-EH1 at the European Synchrotron Radiation Facilities (ESRF) using a cryostream of cold nitrogen (110 K). Processing and scaling were done using the HKL suite of programs (Otwinowski and Minor 1997). Initial phases were obtained by molecular replacement using MOLREP (Vagin and Teplyakov 1997) with the crystal structure of hCHIT1 as a search model (PDB entry 1LG2; Fusetti et al., 2002). Cross-validation (Kleywegt and Brunger 1996) was applied by excluding 1% of the reflections throughout the refinement procedure. Rigid body and simulated annealing followed by several rounds of combined refinement (energy minimization and *B*-factor refinement) using strict noncrystallographic symmetry were done using CNS (Brunger et al., 1998). The graphical program O (Jones et al., 1991) combined with density modification including density averaging from the CCP4 program suite (Collaborative Computational Project 1994) was used for manual adjustments of the structures, and water molecules were included as oxygen atoms after each round of combined refinement using appropriate criteria. Refmac5 (Murshudov et al., 1997) was used in latter stages of refinement. hAMCase crystallized in space group P2_1_2_1_2_1_, and the final models contain six monomers each consisting of 377 residues per protein monomer. The overall fold of the six monomers are similar, with rmsd values (Cα atoms) of 0.21–0.32 Å upon superposition. In the interest of simplicity, the structures are discussed consistently using the first monomer in the coordinate files. Topologies for ligands were obtained using the PRODRG server (Schuttelkopf and van Aalten 2004) and ligands were included using unbiased F_o_-F_c_, ϕ_calc_ electron density maps.

### Generation of AMCase-Expressing Stable Cell Lines

The full-length-coding region of mAMCase was amplified using a lung cDNA template. The cDNA fragment was directionally cloned into pcDNA3.1 (Invitrogen), to generate a V5/His tagged plasmid. TOP10 competent cells were transformed with the AMCase plasmid and sequence confirmed. Mammalian COS-7 cells were transfected with AMCase-pcDNA3.1 plasmid using Lipofectamine 2000 (Invitrogen). COS-7 cell supernatants were screened for AMCase protein by western blot and chitinase activity. A stable AMCase-expressing cell line was generated using G418 selection medium (RPMI).

### Chitinase Activity Assay

Chitinase activity of hAMCase, lung homogenates, or AMCase COS-7 cell supernatants (serum-free) were determined using 4-methylumbelliferyl-β-D-*N,N′,N″*-triacetylchitotrioside and 4-methylumbelliferyl-β-D-*N,N′,N″*-triacetylchitobioside, as described. Samples were incubated with substrate (0.022 mM in 100 mM citric acid, 200 mM sodium phosphate buffer [pH 5.2]) in a final volume of 50 μL. After a 10 min incubation at 37°C, the reaction was stopped with the addition of 500 μl sodium carbonate buffer (0.5 M sodium carbonate and 0.5 M sodium bicarbonate [pH 10.6]). Liberated 4-methlyumberlliferone was quantified using a microplate fluorometer (excitation 360 nm/emission 440 nm). K_i_ and IC_50_ values were determined in the presence of different concentrations of inhibitor. Experiments were performed in duplicate or triplicate.

### OVA Sensitization and Challenge

All experiments used female BALB/c mice, 6- to 8-weeks-old. Mice were kept in individually ventilated cages and all experiments were conducted under UK Home Office guidelines. Mice were sensitized (day 0) and boosted (day 14) i.p. with 20 μg OVA (Grade V, Sigma) adsorbed to 9% potassium alum. Mice were either challenged on day 28 and 30 with 50 μg OVA or PBS by the intratracheal route or were challenged with 1% OVA or PBS by aerosol for 30 min on day 28 to day 30. Bisdionin F or vehicle (2% DMSO in PBS) was administered i.p. 2 hr prior to each challenge at doses indicated in the text. Necropsies were performed 24 hr after the final airway challenge.

### Ventilatory Function

On day 30, prior to the final OVA challenge and bisdionin F dose, ventilatory function (enhanced pause [penH]) was measured using unrestrained whole-body plethysmography (Buxco Systems) and analyzed with system XA software (Buxco Electronics) as previously described (Hamelmann et al., 1997). Briefly, conscious mice were placed in individual chambers for a 10 min acclimatization period. Spontaneous breathing patterns in mice relate to changes in chamber air pressure, which are measured by a transducer attached to the chamber wall. Differences in the rates of pressure change during peak inspiration and peak expiration and the timing of expiration are used to calculate penH according to the following equation; penH = ((expiratory time/relaxation time) – 1) × (peak expiratory flow (mL/s)/peak inspiratory flow (ml/s)). PenH measurements are not used as a quantitative measurement that relates to airway size and rather reflect changes to ventilation following bronchoconstriction. Baseline measurements of penH were made following an aerosol of PBS. Doubling doses of methacholine (3.125→50 mg/mL in PBS, Sigma) were aerosolized for 2 min followed by 5 min data collection. PenH measurements were averaged for the entire dose period.

### Bronchoalveolar Lavage

Approximately 24 hr after the last challenge, mice were killed, the trachea cannulated, and internal airspaces lavaged with 400 μl 0.25% BSA in PBS followed by three 300 μl washes. Total cell numbers were counted and cytospins prepared for differential cell counts, which were assessed by morphology following Diff Quick staining (Reagena). BALF was centrifuged at 1200 × g and supernatant stored at −20°C for further analysis.

### Lung Protein Homogenates

Protein from dissected lung was homogenized (TissueLyser, QIAGEN) in lysis buffer containing protease cocktail inhibitor (Sigma). Samples were incubated for 20 min on ice, prior to centrifugation 10,000 × g to removed cell debris. Protein amounts were quantified with Coomassie (Bradford) Reagent. Homogenates were stored at −70°C for use in the chitinase activity assay.

### Histology

Following BAL, the right lobe of the lung was fix-perfused with 4% formaldehyde and subsequently processed to paraffin and embedded. Standard H&E staining was performed to assess gross pathology and lung neutrophils were visualized by naphthol AS-D choloracetate esterase staining (Sigma).

### RNA Extraction and Quantitative Real-Time PCR

One part of the left lobe of the lung, removed following BALF, was stored in RNAlater (Ambion) at 4°C for up to 4 weeks. Lung samples were homogenized in RLT lysis buffer using TissueLyser (QIAGEN) and total RNA extracted using RNeasy mini spin columns (QIAGEN). RNA (1 μg) was used for synthesis of cDNA using Moloney murine leukemia virus reverse transcriptase. Relative quantification of genes was carried out by RT-PCR using the Roche Lightcycler, as previously described (Nair et al., 2005). PCR amplication was analyzed using 2^nd^ derivative Maximum alogarithm (LightCycler 480 SW 1.5, Roche) and the expression of the gene of interest was normalized to the housekeeping gene, GAPDH. Primer sequences used were as follows: GAPDH-For ATGACATCAAGAAGGTGGTG, Rev CATACCAGGAAATGAGCTTG; Ym1-For TCACAGGTCTGGCAATTCTTCTG, Rev TTGTCCTTAGGAGGGCTTCCTC; Pro Collagen I-For AACTGGACTGTCCCAACCCC, Rev TCCCTCGACTCCTACATCTTCTG; MIP-1α-For TGCCCTTGCTGTTCTTCTCT, Rev GTGGAATCTTCCGGCTGTAG; MMP-12-For CAATTGGAATATGACCCCCTGT, Rev AGCAAGCACCCTTCACTACAT; and TIMP-1-For GTGGGAAATGCCGCAGAT, Rev GGGCATATCCACAGAGGCTTT.

### Cytokine and Chemokine Secretion from Draining Lymph Node Cell Cultures

The draining thoracic lymph nodes (tLN) were dissected and single-cell suspensions made before being plated out at 1 × 10^6^ cells/mL in 96-well plates (RPMI 1640 supplemented with L-glutamine, penicillin streptomycin, and FCS). Cells were stimulated with 500 μg/mL OVA and incubated for 72 hr at 37°C, 5% CO_2_. MIP-1α levels were measured in cell-free supernatants using a Luminex kit (Invitrogen) and samples were read with a Luminex 100 multiplex bead array system.

### Statistical Analysis

Data are expressed as the mean ± standard error of the mean, with individual numbers indicated for each experiment. Statistical analysis was performed with PRISM 4.0 (Graphpad Software). Differences between groups were determined using a one-way ANOVA with Dunnetts post-hoc test. A p < 0.05 was considered a significant difference.

## Figures and Tables

**Table 1:** Summary of Data Collection and Structure Refinement Statistics for the hAMCase-Bisdionin C and F Complexes

	hAMCase + bisdionin C	hAMCase + bisdionin F
Resolution (Å)	20-2.20 (2.25-2.20)	20-2.25 (2.33-2.25)
Cell dimensions (Å)	145.21 149.07 152.08	144.78 149.19 151.28
Number of unique reflections	161985	154470
Multiplicity	4.3	4.1
R_merge_ (%)	10.1 (69.6)	9.6 (57.7)
I/σ(I)	14.1 (2.7)	14.8 (2.7)
Completeness (%)	99.9 (99.9)	99.6 (99.8)
Number of atoms in refinement	19339	19347
Number of solvent molecules	1057	1119
*R*_work_ (%)	18.1	17.3
*R*_free_ (%)	22.8	21.9
Average protein *B*-factor (Å^2^)	31.3	28.5
Average ligand *B*-factor (Å^2^)^a^	42.0	22.6
Average solvent B-factor (Å^2^)	31.5	29.7
Rmsd bond lengths (Å)	0.022	0.023
Rmsd bond angels (°)	1.86	1.89
Ramachandran plot statistics (%)		
Residues in favored regions	97.7	98.0
Residues in allowed regions	2.1	2.0
Residues in outlier regions	0.1	0.0

Values for the highest resolution shell are shown in parenthesis.

a Calculated from ligand molecules occupying the −1 and −3 subsites.
